# Accurate Compensation of Attitude Angle Error in a Dual-Axis Rotation Inertial Navigation System

**DOI:** 10.3390/s17030615

**Published:** 2017-03-17

**Authors:** Rui Jiang, Gongliu Yang, Rui Zou, Jing Wang, Jing Li

**Affiliations:** 1 School of Instrument Science and Opto-Electronics Engineering, Beihang University, Beijing 100191, China; yanggongliu@buaa.edu.cn (G.Y.); buaazr@buaa.edu.cn (R.Z.); by1217133@buaa.edu.cn (J.W.); buaa_lijing@buaa.edu.cn (J.L.); 2 Inertial Technology Key Laboratory of National Defense Science and Technology, Beihang University, Beijing 100191, China

**Keywords:** dual axis rotation INS (inertial navigation system), coning error, attitude error, shaft swing angle, axis non-orthogonal angle, calibration method

## Abstract

In the dual-axis rotation inertial navigation system (INS), besides the gyro error, accelerometer error, rolling misalignment angle error, and the gimbal angle error, the shaft swing angle and the axis non-orthogonal angle also affect the attitude accuracy. Through the analysis of the structure, we can see that the shaft swing angle and axis non-orthogonal angle will produce coning errors which cause the fluctuation of the attitude. According to the analysis of the rotation vector, it can be seen that the coning error will generate additional drift velocity along the rotating shaft, which can reduce the navigation precision of the system. In this paper, based on the establishment of the modulation average frame, the vector projection is carried out, and then the attitude conversion matrix and the attitude error matrix mainly including the shaft swing angle and axis non-orthogonal are obtained. Because the attitude angles are given under the static condition, the shaft swing angle and the axis non-orthogonal angle are estimated by the static Kalman filter (KF). This kind of KF method has been widely recognized as the standard optimal estimation tool for estimating the parameters such as coning angles (α_1_ , α_2_), initial phase angles (ϕ_1_,ϕ_2_), and the non-perpendicular angle (η). In order to carry out the system level verification, a dual axis rotation INS is designed. Through simulation and experiments, the results show that the amplitudes of the attitude angles’ variation are reduced by about 20%–30% when the shaft rotates. The attitude error equation is reasonably simplified and the calibration method is accurate enough. The attitude accuracy is further improved.

## 1. Introduction

The process of improving the precision of the inertial sensor used in an inertial navigation system (INS) is very difficult. Not only new manufacturing process techniques but also advanced assembly techniques and neoteric original materials are necessary to achieve the higher precision [1,2,3]. This is obviously costly. The dual-axis rotation modulating technique means the precision of an INS can be improved without using higher precision gyros. It is one of the strategies of improving the system precision performance at the system level. The key building blocks of the INS comprise an inertial measurement unit, a turntable, a navigation center processing unit (CPU), a display unit, and a control unit. The inertial measurement unit (IMU) has three gyroscopes and three accelerometers. Because the random drift noise cannot be modulated, normally dithered ring laser gyroscopes are used in the rotation modulating INS [4].

Obviously this is much more economical and convenient than the technique of improving the precision of the inertial sensors in an INS. Therefore, this technology has received wide attention in recent years in marine navigation for achieving high performance and low cost [4]. The North Atlantic Treaty Organization (NATO) including the United States uses this technology widely in naval ships and submarines [5,6].

As integration is essence of inertial navigation, the velocity and the position of a carrier can be obtained by integrating the output signals of the gyros and accelerations. Through the presentable symmetrical rotation around the axis with the IMU, some kinds of errors’ mean values such as the constant drift, asymmetry error of scale factor, etc., can be shifted to sinusoidal form [7,8,9,10]. Through the integration, these parameters can approach to zero. Then, the precision of the system is improved indirectly [11,12,13]. Thus the higher position accuracy can be obtained by the rotating modulation technique without raising the inertial component precision.

All technologies have two sides. Either case rotation or platform rotation creates coning errors. The disadvantage of rotation modulation is that it brings out an additional drift rate caused by the rotating vector, and then the attitude error is produced [14]. The nonconforming angle between the axis of casing rotation or the axis of platform rotation and the sensitive axis of the gyroscope obviously exists. In particular, misalignment will cause attitude error [15]. Another disadvantage of modulating technology is that demodulation is needed to show the real attitude of the carrier. That means that the attitude accuracy processing equation is more complex in dual-axis rotation modulation INS than in the strap down inertial navigation system (SINS) and single-axis rotation modulation INS. The attitude transformation matrix considered regarding the nominal trajectory of platform movement, case rolling non-alignment angle, gimbal-angle error, and setting error has been built in [14]. The matrix is suitable for space-stable systems, especially electrostatically suspended gyroscope (ESG) monitors. For dual-axis rotation modulation INS, the attitude transformation matrix should be rebuilt. This has not discussed completely up to now.

The attitude error propagation equation in [16] is concentrated by the fixing error angle between the two axes. A calibration method based on the theoretical study is put forward in this article. A demodulation program has been built regarding the angle conversion between the six different coordinate systems. The angle between the IMU and inner axis and the angle between the inner axis and outer axis are calculated by traditional calibration. [17] discusses the influence of the installation inclination angle on attitude accuracy. Because the analysis is based on the single-axis system, only the azimuth angle is considered. An approximate sinusoidal principle mathematic model is established and applied to the compensation of the vehicle attitude output deviation. [18] provides the error model considering the gyro drift, accelerometer error, misalignment angle of the gyro case rotation axis, installation error, and frame angle bias. The system parameters are identified by the least squares identification method. The discussion focuses on the attitude accuracy of space-stable INS. [19] builds the calibration and compensation method for the precision of the output attitude in the dual-axis rotational INS. The mounting errors between the inertial measurement unit and the turntable are analyzed and calibrated. The calibration method is a thin-shell (TS) algorithm. Again, the turntable frame is an orthogonal coordinate frame. Only article [20] talks about the redundancy angle variation causing the cross coupling effect. It can also cause velocity and attitude disturbances. The model is still based on the coordinate transformation between the platform frame and ESG.

All the articles mentioned above do not analyze the axis non-orthogonal angle. The research done before also does not consider the coupled effects caused by this angle. Therefore, the attitude error caused by the shaft swing angle cannot be compensated thoroughly. The variation of the velocity and attitude are increased with the increase of the axis non-orthogonal angle. This article puts emphasis on the non-orthogonal angle of the axes and the shaft swing angle among all the diathesis.

The structure diagram is used to illustrate that these two angles are indeed unavoidable. The variation component of the shaft swing angle will increase the uncertainty component in the attitude error. Observing the actual uncompensated attitude of the designed dual-axis rotational system, two main kinds of error are shown. One has a sinusoidal curve, and the other has a jagged curve. The quantity of the former is about scores of arc-min and the latter is about 10% of the former. As for the sinusoidal curve, the core of the theory is the coning error which is evidenced by the rotating vector differential equation. Besides, the rolling non-alignment angle, axis non-orthogonal angle, and shaft swing angle all influence the attitude transform matrix through coining error formation. So the attitude precision is determined greatly by these factors. It also decides the platform price of mainly the two axis platform. The cross coupling effect due to shaft swing angle and its variation should make the platform rolling non-alignment angle not be compensated completely. Due to the axis non-orthogonal angle, periodic components exist. When the IMU rotates around the horizontal axis, the X gyro and Y gyro will produce periodic components on the horizontal axis. In order to obtain high precision attitude, we need to determine the axis non-orthogonal angle and the shaft swing angle. Then we can compensate for them.

The direction-cosine matrix (DCM) including the axis non-orthogonal angle and platform rolling angle is described based on the geometric projection of the error vectors. In order to obtain the values of the parameters, the calibration and compensation methods are given based on the attitude error matrix. Notice that the attitude angle and heading angle are unchanged under the static condition, and the fluctuation of the attitude angle and heading angle can be used as observations. Five resolutions of vectors which are decomposed by the non-orthogonal angle and shaft swing angle in the navigation coordinate can be taken as state vectors. Then, the observed equation is developed in this article. 500 Monte Carlo simulations are performed to verify the filtering accuracy. Simulation and test results show that, with the new compensation model, the attitude errors can be reduced by about three or four times.

The outline of this paper is as follows: Section 1 is the introduction. The brief description about the gimbaled structure of the dual-axis rotation inertial navigation system is introduced briefly in Section 2, and the cause of the non-orthogonal angle and the shaft swing angle is also given in this section. Section 3 provides the definition of correlative coordinates. The modulation average frame is especially defined here to help set up the projection of the axis non-orthogonal angle. The expression form and the influence of the coning error are explained in detail by the rotating vector differential equation in Section 4, and the attitude transform matrix and attitude error equation are built in this section. In Section 5, the calibration method is shown. The simulation results and the practical experiment results are shown in Section 6 along with the discussion. The conclusions are given in Section 7 with some necessary discussions.

## 2. Structure of the Dual-Axis

**Figure 1:**
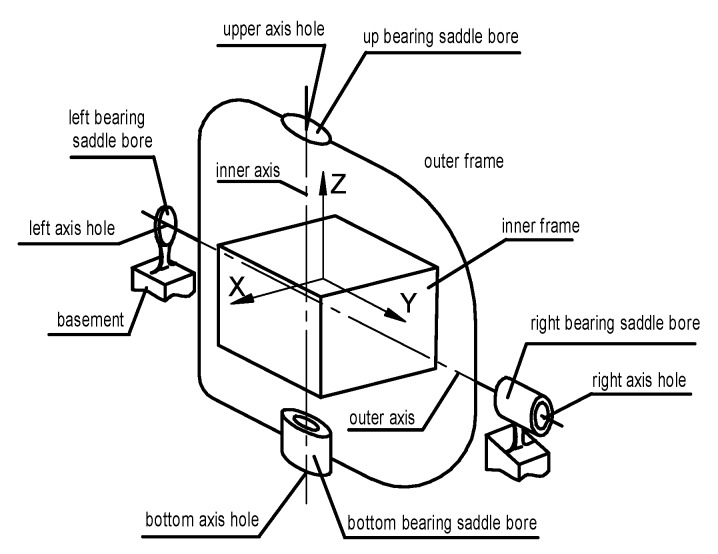
Structural diagram of the dual-axis rotational inertial navigation system.

The axes of the dual-axis rotational inertial navigation system should be orthogonal in space. The structure of the dual-axis rotational INS is shown in Figure 1.

Notice that any one of the axes consists of two discontinuous parts due to the IMU. The figure shows that the inner axis is neither the upper axis nor the lower axis. The communal axis which is determined by the upper and lower axes is actually the real rotational axis which is not any one of the physical axes. It is the same as the outer axis. The outer axis is the communal axis determined by the left and right axis. This means the center lines’ positions of the rotary axes are determined by the axis hole and the axis itself. The swing amplitude of the axis is affected by the coaxial degree between the shaft hole, the bearing saddle bore, and the bearing precision. The verticality of the common axes of the two sets of holes on the outer gimbal and the coaxiality of the two holes on the inner gimbal influence the initial static perpendicularity of the inner axis and the outer axis. The vertical and lateral degrees of the inner and outer axes are affected by the common axis, each of which consists of two holes on the outer gimbal. There are many factors that determine the stability of the actual axis, such as the machining precision of the holes, the precision of the bearings, the assembly precision, etc. As a result, the inner axis cannot be perpendicularly rigid with the outer axis. Even the co-planarity requirement of the two axes is difficult to ensure. So the actual rotation axis is swaying all the time when the physical axis is rotating.

The turntable frame is a non-orthogonal coordinate system because of the non-perpendicularity of the two rotational axes. Furthermore, the rotational axes are unstable with the rotation of the shaft. The non-orthogonal angle between the inner axis and the outer axis and the swaying of each communal axis introduce additional errors of the attitude. A quantitative analysis in theory is necessary to determine the effects of the orthogonal error and the swaying error and whether to compensate for such errors.

## 3. Coordinate Systems

The error analysis is developed in the determined orthogonal and right-handed coordinate frames. However, it is obvious that the sensitive axes of gyros and accelerometers are not orthogonal in the actual physical structures [19]. In addition, the turntable frame is also non-orthogonal. All useful frames should be established for modeling and analysis.

**Figure 2:**
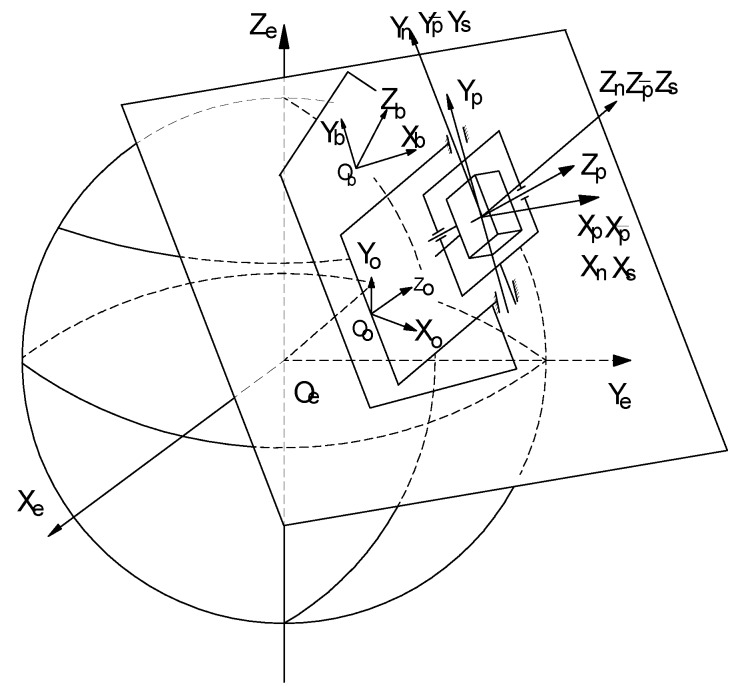
Regulation of the different frames.

The frames include the IMU, turntable, modulation average, basement, the body, and navigation frames. Figure 2 shows the relationship between these when the INS is settled on a horizontal plane.

### 3.1. IMU Frame

The IMU frame, denoted by S, is an orthogonal coordinate frame. All the accelerometers and gyros are not strictly orthogonally mounted. For ensuring correct calculations, all sensor outputs should be transformed into an orthogonal coordinate frame called the IMU frame. The origin of this frame is the centroid of the IMU. At the beginning time, the Y_s_ is defined to coincide with the Y_g_. The X_s_ is perpendicular with the Y_s_ in the same plane. The Z_s_ axis is orthogonal to both X_s_ and Y_s_, which make up a right-hand orthogonal frame. Because the non-orthogonal angles between the gyros and accelerometers are not considered here, the errors between the IMU frame, gyro frame, and accelerometer frame are considered as zero. The S frame is strapped to the turntable and rotates with it. At the beginning time, the S frame coincides with the n frame. At the same time, the angles of the two encoders mounted on the gimbals are marked as zero.

### 3.2. Turntable Frame

The turntable frame, denoted by P, is defined by the turntable’s two axes as a real frame. The Y_p_ axis is defined to coincide with the direction of the outer axis of the turntable. The Z_p_ axis is consistent with the inner axis of the turntable. The X_p_ axis is orthogonal to both Y_p_ and Z_p_ axes and makes up a right-hand orthogonal frame. The P frame is a non-orthogonal frame because the Z_p_ axis is not perpendicular to the Y_p_ axis. This frame can be also expressed as {Y_p_ × Z_p_ ,Y_p_, Z_p_} Of course, it is a dynamic coordinate system.

### 3.3. Modulation Average Frame

The modulation average frame, denoted by $\bar{P}$, is an orthogonal coordinate system. Since the P frame is non-orthogonal, it is necessary to build a new orthogonal frame so that the navigation parameter resolution can be calculated in this frame. In order to simplify the calculation and using the projection method, we define the $Z_{\bar{P}}$ axis, the $X_{\bar{P}}$ axis overlap with the Z_p_ axis, and the X_p_ axis, respectively, when the P frame is in the initial state. The $Y_{\bar{P}}$ axis is orthogonal to both $X_{\bar{P}}$ and $Z_{\bar{P}}$ axes and makes up a right-hand orthogonal frame. This frame can be also expressed as {Y_p_ × Z_p_ , Z_p_ × (Y_p_ × Z_p_), Z_p_}. The misalignment angle between the $\bar{P}$ frame and the n frame is considered as zero to simplify the calibration. This frame is a dynamic coordinate system rotating with the turntable.

### 3.4. Body Frame

The body frame, denoted by b, is rigidly attached to the vehicle. The X_b_ axis points rightward, the Y_b_ axis points forward, and the Z_b_ axis points upwards. The origin of this frame is in the centroid of the vehicle. In the initial state, the body frame coincides with the $\bar{P}$ frame.

### 3.5. Navigation Frame

The navigation frame, denoted by n, is defined by the East-North-Up geographic frame. The X_n_ axis points in the direction of geodetic east. The Y_n_ axis points in the direction of geodetic north. The Z_n_ axis points in the direction of geodetic vertical. The attitude of the ship is defined by the Euler angles of the body frame with respect to the corresponding navigation frame.

## 4. Coning Error Analysis and Attitude Modeling

According to the definition of all the frames, the S, $\bar{P}$, b, n frames are all orthogonal coordinate frames. Only the P frame is a non-orthogonal coordinate frame. After the rolling misalignment angle of the S frame with the P frame has been calibrated and compensated, the shaft swing angle caused by the bearing accuracy and shaft assembly accuracy and the non-orthogonal angle between the $\bar{P}$ frame and P frame produce coning errors.

### 4.1. Coning Error Analysis

**Figure 3:**
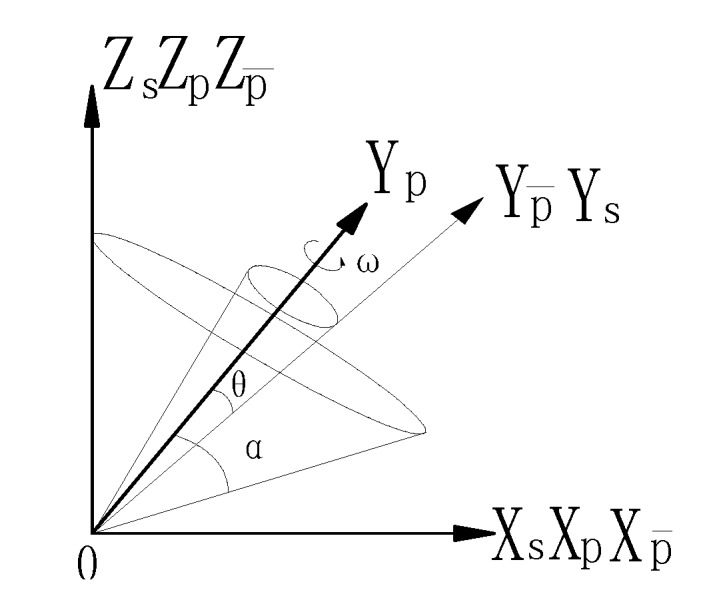
Coning error caused by the non-orthogonal angle of the P frame.

When the S frame rotates around the oy_p_ axis, two cones are created according to the definition of the frames. The ${\mathrm{oy}}_{\bar{p}},{\mathrm{oz}}_{\bar{p}}$ axes are the generatrices of the two cones, respectively. The oy_p_ axis is the symmetrical axis. Figure 3 shows the two cones.

The rotation vector from the S frame to the P frame is expressed as

(1) $$\varphi_{\mathrm{ps}}^{s}={\left[\begin{array}{ccc} \phi_{x}^{s} & \phi_{y}^{s} & \phi_{z}^{s} \end{array}\right]}^{\prime }=\phi u$$

where, $u=\frac{\varphi }{\phi }$ is a unit column vector along the direction of the rotational axis. The orientation here is the direction of the oy_p_ axis;

$\phi =\sqrt{{\left(\phi_{x}^{s}\right)}^{2}+{\left(\phi_{y}^{s}\right)}^{2}+{\left(\phi_{z}^{s}\right)}^{2}}$. The rotation angle φ is created when the S frame rotates around the axis of the P frame;

When the rotational velocity is ω, the relationship between the rotation vector **ϕ** and the rotational velocity vector **ω** can be expressed as

(2) $$\overset{\cdot }{\varphi }=\omega +\frac{1}{2}\varphi \times \omega +\frac{1}{\phi^{2}}(1-\frac{\phi }{2}\cos(\frac{\phi }{2}))\varphi \times (\varphi \times \omega )$$

The first item on the right is an interchangeable component. It is a fixed axis rotation component. The second term is the cone component. The cone movement of the gyroscope sensitive axis is caused by the rolling non-alignment angle, shaft swing angle, and axis non-orthogonal angle. This kind of cone motion creates a certain drift angular speed along the axis of the rotation direction. The expression obtained by matrix inverse for (2) is

(3) $$\omega =\left\{I-\frac{1-cos\phi }{\phi^{2}}\left[\varphi \right]+\frac{1}{\phi^{2}}\left(1-\frac{sin\phi }{\phi }\right){\left[\varphi \right]}^{2}\right\}\overset{\cdot }{\varphi }$$

where **ω** is the additional drift angular velocity, which increases the attitude errors. As a result, the rate of roll angle error is increased. It is easy to see from Figure 3 that two gyroscopes produce the coning movement after the rolling non-alignment angle has been calibrated. The gyro with the sensitive axis that is coincident with the rotational axis produces the drift angular velocity directly. The drift angular velocity caused by the other gyro couples to the rotational axis. Not only the attitude error but also the position error increases. The coupling component of the cone motion, created by the vertical gyro rotating around the oy_p_ axis, causes the fluctuation of the roll angle. Therefore, it is necessary to calibrate and compensate for the non-orthogonal angle and the shaft swing angle to improve the attitude precision.

Article [17] introduces a calibration method to calibrate the skew angles, which are always large angles. This article focuses on the small angles after large coning errors have been compensated for.

### 4.2. Attitude Modeling Method

In order to obtain the relatively complete formula of the attitude, some transformation matrices should be established.

#### 4.2.1. The Transformation Matrix between the S Frame and the $\bar{P}$ Frame

**Figure 4:**
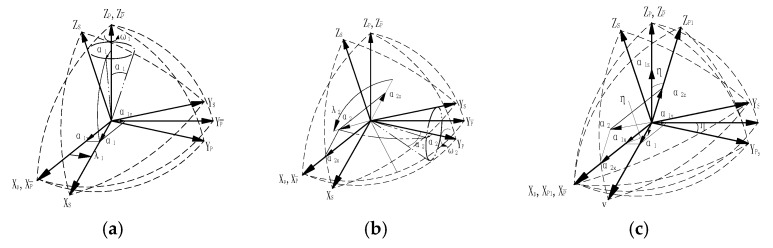
Geometric projection of the shaft swing angle and the non-orthogonal angle between the S frame, P frame, P_1_ frame, and the $\bar{P}$ frame. (**a**) one cone created when IMU rolls around the oz_p_ axis; (**b**) another cone when IMU rolls around the oy_p_ axis; (**c**) the geometric projection of the non-orthogonal angle in the $\bar{P}$ frame.

The figure of the relationship between the S frame and the $\bar{P}$ frame is shown in Figure 4 according to the definition mentioned in Section 3.

Here we assume that the misalignment angle between the S frame and the P frame has been calibrated and compensated for. The emphasis here is put on the shaft swing angle and the non-orthogonal angle, which are small angles and are caused by the reasons mentioned in Section 2. In Figure 4c, η is a non-orthogonal angle between the oz_p_ axis and the oy_p_ axis, and is also the angle between the oy_p_ axis and the $oy_{\bar{p}}$ axis. In Figure 4a, α_1_ is a cone angle caused when the S frame rotates around the oz_p_ axis. In Figure 4b α_2_ is a cone angle caused when the S frame rotates around the oy_p_ axis. α_1_ and α_2_ belong to the shaft swing angle. The rotational angular velocity of the oz_p_ axis and oy_p_ axis are ω_1_ and ω_2_, respectively. So the shaft swing angle along the oz_p_ axis can be resolved as:

(4) $$\alpha_{1x}=\alpha_{1}\cos\lambda_{1}$$

(5) $$\alpha_{1y}=\alpha_{1}\sin\lambda_{1}$$

where λ_1_ = λ + ϕ_1_, and λ is the rotation angle when the IMU rotates around the oz_p_ axis. It can be measured by the angle encoder or calculated as λ=ω_1_t. ϕ_1_ is the initial phase angle.

Similarly, the shaft swing angle along the $oy_{P}$ axis can be written as:

(6) $$\alpha_{2x}=-\alpha_{2}\sin\lambda_{2}$$

(7) $$\alpha_{2z}=\alpha_{2}\cos\lambda_{2}$$

where λ_2_ = λ + ϕ_2_, and ϕ_2_ is the initial phase angle. Generally, ω_1_ = ω_2_ = ω. Then λ = ω_1_t = ω_2_t = ωt.

Three angles need to rotate to transform the S frame to the $\bar{P}$ frame. First, the S frame rotates around the $oz_{\bar{P}}$ axis with α_1z_; Second, the S^’^ frame rotates around the oy_s_ axis with α_1y_; Third, the S^”^ frame rotates around the ox_s_ axis with α_1x_. Notice that the non-orthogonal angle affects the computing coning angle when we obtain the DCM between the S frame and the $\bar{P}$ frame. The P_1_ frame is created by rotating the $\bar{P}$ frame around the X_p_ axis with angle η to help find its projection in the $\bar{P}$ frame. The geometric projection of the non-orthogonal angle in the $\bar{P}$ frame is shown in Figure 4c. So the relationship between α_1z_ and η is

(8) $$\alpha_{1z}\cos\eta =\alpha_{2z}-\alpha_{1x}\sin\eta$$

By substituting Equation (8) with Equations (4) and (7),

(9) $$\alpha_{1z}\cos\eta =\alpha_{2}\cos\lambda_{2}-\alpha_{1}\cos\lambda_{1}\sin\eta$$

since

(10) $$C_{S}^{\bar{P}}={\left(C_{\bar{P}}^{S}\right)}^{T}=\left[\begin{array}{ccc} 1 & -\alpha_{1z} & \alpha_{1y}\\ \alpha_{1z} & 1 & -\alpha_{1x}\\ -\alpha_{1y} & \alpha_{1x} & 1 \end{array}\right]$$

so

(11) $$C_{S}^{\bar{P}}=\left[\begin{array}{ccc} 1 & -\alpha_{2}\omega \cos\lambda_{2}\sec\eta +\alpha_{1}\omega \cos\lambda_{1}\tan\eta & \alpha_{1}\omega \sin\lambda_{1}\\ \alpha_{2}\omega \cos\lambda_{2}\sec\eta -\alpha_{1}\omega \cos\lambda_{1}\tan\eta & 1 & -\alpha_{1}\omega \cos\lambda_{1}\\ -\alpha_{1}\omega \sin\lambda_{1} & \alpha_{1}\omega \cos\lambda_{1} & 1 \end{array}\right]$$

and

(12) $$\alpha ={\left[\begin{array}{ccc} \alpha_{1}\cos\lambda_{1} & \alpha_{1}\sin\lambda_{1} & \alpha_{2}\cos\lambda_{2}\sec\eta -\alpha_{1}\cos\lambda_{1}\tan\eta \end{array}\right]}^{T}$$

#### 4.2.2. Attitude Model Method

The attitude angle should be demodulated because of the rotation of the IMU. The real output of the body can be calculated from the angles between the IMU frame and the navigation frame by removing the turntable’s rotation angles from the IMU attitude. We can obtain more precise attitudes if the parameters such as α_1_, α_2_, and η mentioned before are calibrated and compensated for correctly. The attitude transform matrix $C_{b}^{n}$ is important in the process of calculating the attitude of the dual-axis rotation INS. All useful and necessary messages for the attitude algorithm are included in this matrix. The rotation from the b frame to the n frame can be derived from three rotations, $C_{\bar{P}}^{n}$, $C_{S}^{\bar{P}}$, $C_{b}^{S}$, using the relation

(13) $$C_{b}^{n}=C_{\bar{P}}^{n}C_{S}^{\bar{P}}C_{b}^{S}$$

where $C_{\bar{P}}^{n}$ is a transform matrix from the $\bar{P}$ frame to the n frame. This matrix can be decomposed into two matrices.

(14) $$C_{\bar{P}}^{n}=C_{\bar{P_{0}}}^{n}C_{\bar{P}}^{\bar{P_{0}}}$$

It does not matter whether the rotation scheme is 8, 16, or 64 sequences; the turntable rotates among four static positions in absolutely symmetrical sequences. Therefore, the turntable attitude matrix $C_{\bar{P}}^{n}$ at the four positions 1, 2, 3, and 4 can be written as:

(15) $$\begin{array}{l} {\left(C_{\bar{P}}^{\bar{P_{0}}}\right)}_{1}=\left[\begin{array}{ccc} 1 & 0 & 0\\ 0 & 1 & 0\\ 0 & 0 & 1 \end{array}\right]\begin{array}{cc} & {\left(C_{\bar{P}}^{\bar{P_{0}}}\right)}_{2}=\left[\begin{array}{ccc} -1 & 0 & 0\\ 0 & -1 & 0\\ 0 & 0 & 1 \end{array}\right] \end{array}\\ {\left(C_{\bar{P}}^{\bar{P_{0}}}\right)}_{3}=\left[\begin{array}{ccc} 1 & 0 & 0\\ 0 & -1 & 0\\ 0 & 0 & -1 \end{array}\right]\begin{array}{cc} & {\left(C_{\bar{P}}^{\bar{P_{0}}}\right)}_{4}=\left[\begin{array}{ccc} -1 & 0 & 0\\ 0 & 1 & 0\\ 0 & 0 & -1 \end{array}\right] \end{array} \end{array}$$

(16) $$C_{\bar{P_{0}}}^{n}=\left[\begin{array}{ccc} -\sin L & 0 & \cos L\\ 0 & 1 & 0\\ -\cos L & 0 & -\sin L \end{array}\right]$$

$C_{b}^{S}$ is the transformation matrix from the body frame b to the turntable frame S. It can be represented by two angles measured by the two angle encoders in real-time. β is the relative angle between the inner gimbal and the outer gimbal. ζ is the relative angle between the outer gimbal and the basement. The expression is written as:

(17) $$\begin{array}{ll} C_{b}^{S} & =\left[\begin{array}{ccc} \cos(-\beta ) & \sin(-\beta ) & 0\\ -\sin(-\beta ) & \cos(-\beta ) & 0\\ 0 & 0 & 1 \end{array}\right]\left[\begin{array}{ccc} \sin(-\zeta ) & 0 & -\cos(-\zeta )\\ 0 & 1 & 0\\ \cos(-\zeta ) & 0 & \sin(-\zeta ) \end{array}\right]\\ & \begin{array}{c} =\left[\begin{array}{ccc} -\cos\beta \sin\zeta & -\sin\beta & -\cos\beta \cos\zeta \\ -\sin\beta \sin\zeta & \cos\beta & -\cos\zeta \sin\beta \\ \cos\zeta & 0 & -\sin\zeta \end{array}\right] \end{array} \end{array}$$

$C_{b}^{n}$ represents the DCM of the n frame with respect to the b frame. When the yaw, pitch, and roll angle are ψ, θ and Υ, respectively, the $C_{b}^{n}$ can be written as:

(18) $$\begin{array}{ll} C_{b}^{n} & =\left[\begin{array}{ccc} \cos\gamma \cos\psi +\sin\gamma \sin\psi \sin\theta & \sin\psi \cos\theta & \sin\gamma \cos\psi -\cos\gamma \sin\psi \sin\theta \\ -\cos\gamma \sin\psi +\sin\gamma \cos\psi \sin\theta & \cos\psi \cos\theta & -\sin\gamma \sin\psi -\cos\gamma \cos\psi \sin\theta \\ -\sin\gamma \cos\theta & \sin\theta & \cos\gamma \cos\theta \end{array}\right]\\ & \begin{array}{c} =\left[\begin{array}{ccc} T_{11} & T_{12} & T_{13}\\ T_{21} & T_{22} & T_{23}\\ T_{31} & T_{32} & T_{33} \end{array}\right] \end{array} \end{array}$$

So

(19) $$\Phi ={\left[\begin{array}{ccc} \theta & \gamma & \psi \end{array}\right]}^{T}={\left[\begin{array}{ccc} \arcsin T_{32} & -\arctan\frac{T_{31}}{T_{32}} & \arctan\frac{T_{12}}{T_{22}} \end{array}\right]}^{T}$$

### 4.3. Attitude Error Modeling Method

Substituting Equation (14) into (13) and differentiating both sides of the equation gives:

(20) $$\delta C_{b}^{n}=\delta C_{\bar{P_{0}}}^{n}C_{\bar{P}}^{\bar{P_{0}}}C_{S}^{\bar{P}}C_{b}^{S}+C_{\bar{P_{0}}}^{n}C_{\bar{P}}^{\bar{P_{0}}}\delta C_{S}^{\bar{P}}C_{b}^{S}+C_{\bar{P_{0}}}^{n}C_{\bar{P}}^{\bar{P_{0}}}C_{S}^{\bar{P}}\delta C_{b}^{S}$$

where,

(21) $$\delta C_{b}^{n}=-\left[\Phi^{n}\right]C_{b}^{n}=-\left[\begin{array}{ccc} 0 & \phi_{\psi } & -\phi_{\gamma }\\ -\phi_{\psi } & 0 & \phi_{\theta }\\ \phi_{\gamma } & -\phi_{\theta } & 0 \end{array}\right]C_{b}^{n}$$

Φ^n^ is the attitude angle error vector. It can be expressed as

(22) $$\Phi^{n}={\left[\begin{array}{ccc} \phi_{\theta } & \phi_{\gamma } & \phi_{\psi } \end{array}\right]}^{T}$$

(23) $$\begin{array}{l} \delta C_{S}^{\bar{P}}=-\left[\overset{g}{\alpha }\right]C_{S}^{\bar{P}}\\ =\left[\begin{array}{ccc} 0 & \alpha_{2}\omega \cos\lambda_{2}\sec\eta -\alpha_{1}\omega \cos\lambda_{1}\tan\eta & -\alpha_{1}\omega \sin\lambda_{1}\\ -\alpha_{2}\omega \cos\lambda_{2}\sec\eta +\alpha_{1}\omega \cos\lambda_{1}\tan\eta & 0 & \alpha_{1}\omega \cos\lambda_{1}\\ \alpha_{1}\omega \sin\lambda_{1} & -\alpha_{1}\omega \cos\lambda_{1} & 0 \end{array}\right]C_{S}^{\bar{P}} \end{array}$$

(24) $$\delta C_{b}^{S}=-\left[\delta \chi^{S}\right]C_{b}^{S}=-\left(\left[\begin{array}{c} 0\\ \delta \zeta \\ 0 \end{array}\right]+\left[\begin{array}{ccc} \cos\zeta & 0 & \sin\zeta \\ 0 & 1 & 0\\ -\sin\zeta & 0 & \cos\zeta \end{array}\right]\left[\begin{array}{c} 0\\ 0\\ -\delta \beta \end{array}\right]\right)C_{b}^{S}$$

(25) $$\delta \chi ={\left[\begin{array}{ccc} \delta \beta \sin\zeta & -\delta \zeta & \delta \beta \cos\zeta \end{array}\right]}^{T}$$

Substituting Equations from (21) to (25) into Equation (20), and then right multiplying $C_{n}^{b}$ on both sides of the equation. At last changing the result to vector form:

(26) $$\Phi^{n}=C_{\bar{P}}^{n}\overset{\cdot }{\alpha }+C_{S}^{n}\chi$$

Because the gimbal angle error is not the main error considered here, Equation (26) can be rewritten as

(27) $$\Phi^{n}=C_{\bar{P}}^{n}\overset{\cdot }{\alpha }$$

So

(28) $$\left[\begin{array}{c} \phi_{\theta }\\ \phi_{\gamma }\\ \phi_{\psi } \end{array}\right]=\left[\begin{array}{ccccc} \sin(\omega t-\beta ) & \cos(\omega t-\beta ) & 0 & 0 & 0\\ \sin L\cos(\omega t-\beta ) & \sin L\sin(\omega t-\beta ) & -\cos L\sin\omega t & \cos L\cos\omega t & \sin L\cos\omega t\\ \cos L\cos(\omega t-\beta ) & \cos L\sin(\omega t-\beta ) & \sin L\sin\omega t & -\sin L\cos\omega t & \cos L\sin\omega t \end{array}\right]\cdot \left[\begin{array}{c} \alpha_{1}\omega \cos\phi_{1}\\ \alpha_{1}\omega \sin\phi_{1}\\ \alpha_{2}\omega \cos\phi_{2}\sec\eta \\ \alpha_{1}\omega \cos\phi_{1}\tan\eta \\ \alpha_{2}\omega \sin\phi_{2} \end{array}\right]$$

According to Equation (20), it is obvious to see that the attitude errors are caused by the non-orthogonal angle, the shaft swing angle, and the gimbal angle error. The amplitude of the attitude errors directly increase with the increase of the coning angles α_1_, α_2_, and the non-orthogonal angle η.

## 5. Calibration Method

Before calibrating the model parameters of the shaft swing angle (α_1_, ϕ_1_), (α_2_, ϕ_2_), and the non-orthogonal angle η, traditional methods should be used to calibrate and compensate for the IMU errors in the conventional sensor model. Then the mounting errors should be calibrated using the method mentioned in article [17].

The parameters (α_1_, ϕ_1_), (α_2_, ϕ_2_), and η can be calibrated by using the difference between the in-situ test data and the known attitude angle. The roll and pitch angles are zero when the system is set on a horizontal basement. At the same time, the head angle is a fixed value. We can use this information to calibrate the parameters by the Kalman filter.

The state vectors of the filter can be set as:

(29) $$X=\left[\begin{array}{ccccc} \alpha_{1}\cos\phi_{1} & \alpha_{1}\sin\phi_{1} & \alpha_{2}\cos\phi_{2}\sec\eta & \alpha_{1}\cos\phi_{1}\tan\eta & \alpha_{2}\sin\phi_{2} \end{array}\right]$$

The measurement vectors are

(30) $$Z_{k}={\left[\begin{array}{ccc} \phi_{x} & \phi_{y} & \phi_{z} \end{array}\right]}^{T}$$

From Equation (28), the measurement equation is

(31) Z=Hx+υ

where H is the measurement matrix.

$$H=\left[\begin{array}{ccccc} \sin(\omega t-\beta ) & \cos(\omega t-\beta ) & 0 & 0 & 0\\ \sin L\cos(\omega t-\beta ) & \sin L\sin(\omega t-\beta ) & -\cos L\sin\omega t & \cos L\cos\omega t & \sin L\cos\omega t\\ \cos L\cos(\omega t-\beta ) & \cos L\sin(\omega t-\beta ) & \sin L\sin\omega t & -\sin L\cos\omega t & \cos L\sin\omega t \end{array}\right]$$

υ is the measurement noise that has the nature of white noise. It satisfies

(32) $$\begin{array}{ccc} E(\upsilon )=0 & & E(\upsilon_{k}{\upsilon_{j}}^{T})=R_{k}\delta_{\mathrm{kj}} \end{array}$$

where R is a symmetic, positive, definite matrix. $\delta_{\mathrm{kj}}=\{\begin{array}{ccc} 1 & & k=j\\ 0 & & k?j \end{array}$ is the Kronecher δ function.

Suppose that x_0_ has $\bar{x_{0}}$ as its mean value and P_0_ as the covariance matrix is independent with {υ}. Thus, the state Kalman filter is

(33) $$\begin{array}{l} \begin{array}{ccc} \hat{x}(t_{k})=\hat{x}(t_{k-1})+P(t_{k})H(t_{k})R^{-1}(z(t_{k})-H(t_{k})\hat{x}(t_{k-1})), & & \hat{x}(t_{0})={\bar{x}}_{0} \end{array}\\ \begin{array}{ccc} P(t_{k})={\left(P(t_{k-1})+H(t_{k})R^{-1}H^{T}(t_{k})\right)}^{-1}, & & P(t_{0})=P_{0} \end{array} \end{array}$$

According to the measurement vector z(t_k_), the filter state estimation vector $\hat{x}(t_{k})$ can be obtained. When the output of the filter is stable, the estimated variables converge to the true value, and the calibration process is over. After obtaining the estimate of the variables, the shaft swing angle and the non-orthogonal angle can be calculated as follows:

(34) $$\begin{array}{l} {\hat{\alpha }}_{1}=\sqrt{{{\hat{x}}_{1}}^{2}+{{\hat{x}}_{2}}^{2}}\\ {\hat{\phi }}_{1}=\arctan2({\hat{x}}_{1},{\hat{x}}_{2})\\ \eta =\arctan2({\hat{x}}_{4},{\hat{x}}_{1}))\\ {\hat{\alpha }}_{2}=\sqrt{({\hat{x}}_{3}\cos{\left(\arctan2({\hat{x}}_{4},{\hat{x}}_{1})\right)}^{2}+{{\hat{x}}_{5}}^{2}}\\ {\hat{\phi }}_{2}=\arctan2({\hat{x}}_{5},{\hat{x}}_{3}\cos(\arctan2({\hat{x}}_{4},{\hat{x}}_{1}))) \end{array}$$

## 6. Results

### 6.1. Simulation Results

To verify the compensation effect of the method of calibrating the rolling non-alignment angle and the non-orthogonal angle, a simulation experiment was performed. The data used in the simulation were collected from the designed and manufactured INS mentioned in Section 6. In this system, the IMU is an independent unit. Not only three dithered ring laser gyroscopes and three quartz accelerometers, but also a data acquisition board and a calculating board are used in the IMU. Therefore, the IMU can output the angular velocity and the acceleration of the vehicle by operating a certain program in Digital Signal Processing board.

In order to guarantee the repeatability of the results, the test environment is kept as consistent as possible. The system preheating time, the data acquisition time, method of data acquisition, test equipment, navigation solution method, the rotation scheme, etc., are all kept the same during this study.

The IMU has been calibrated independently before being mounted in the turntable. The mislignment matrix of the gyro and accelerometer in the IMU frame and the scale factor are all calibrated. The specifications of the designed dual-axis rotation INS are shown in Table 1. Data are collected for 200 s in each test group, with an angular velocity of 10°/s (±0.1%) for the calibration. After all kinds of the mounting errors and the angle errors of the angle encoders are calibrated for, we can use Equation (28) to calibrate for the slight errors such as α, ϕ, and η.

Here a 500 Monte Carlo simulation was used to evaluate the calibration precision. The swaying angle for the calibration was set following the distribution S (−$10^{\prime }$, +$10^{\prime }$). The phase for the calibration was set following the distribution A (−$1^{{}^{\circ}}$, +$1^{{}^{\circ}}$). The non-orthogonal angle for calibration was set following the distribution Q (−20′′, +20′′). Table 2 is a set of simulation results for certain errors. Table 3 shows the precision of the calibration estimated by the 500 Monte Carlo simulation.

### 6.2. Laboratory Experimental Results

**Figure 5:**
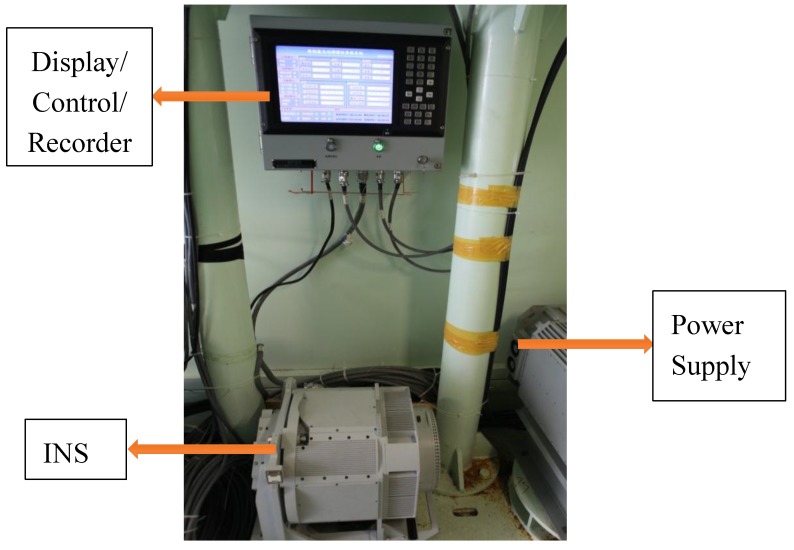
An overall view of the designed dual-axis rotational INS.

A dual-axis rotational inertial navigation system was designed for the experiment, shown in Figure 5. The turntable was designed in theory by two orthogonal gimbals. The IMU was mounted in the middle of the turntable. Driven by the torque motor, the IMU rotates about one axis at one time alternately with a special scheme. In consideration of the calibration in real-time and the isolation of the craft movement [21,22], the inner gimbal rotates in an azimuth plane and the outer gimbal rotates in a horizontal plane. Therefore the inner axis can be called the vertical axis, and the outer axis can be called the rolling axis.

**Figure 6:**
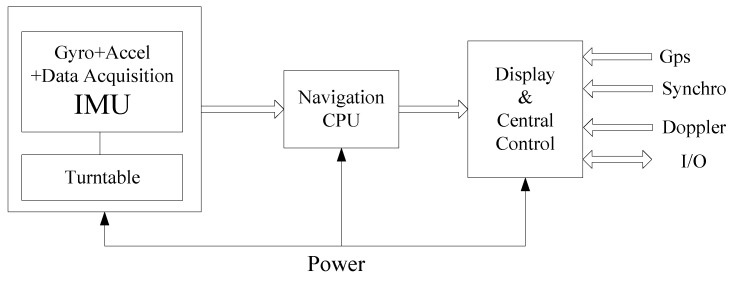
A functional diagram of the designed dual-axis rotational INS.

A navigation grade IMU was developed using three dithered ring laser gyroscopes (0.005°/h) and three quartz accelerometers (100 μg). On the rotors of the gimbals, two electrical torques and two angle encoders were mounted to control the rotations. In addition, three slip-rings were mounted individually in the up-axis, down-axis, and right-axis. They support cables connecting the inertial sensors and outer circuits. Figure 6 shows the functional diagram of the system.

#### 6.2.1. The Data Analysis before Compensation

**Figure 7:**
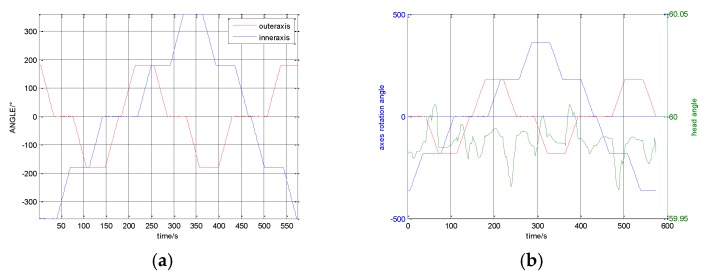

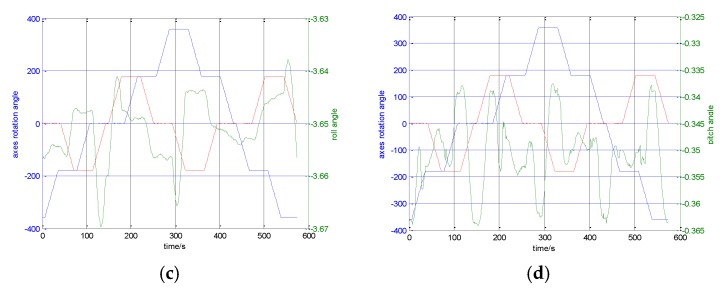
System attitude angle after compensating for the misalignment angle. (**a**) Two axes rolling angels; (**b**) Head angle; (**c**) Roll angle; (**d**) Pitch angle.

After compensating for the installation misalignment angle, the attitude of the INS is shown in Figure 7.

Figure 7a shows the rotation angles and the sequence of the two axes. The head angle change regulation of the head angle with the axes is shown in Figure 7b. The change regulation of the roll angle with the axes is shown in Figure 7c. The change regulation of the pitch angle with the axes is shown in Figure 7d. Observing these curves, it can be found that the head angle changes with the inner axis rotation. The roll angle changes with the outer axis rotation. Any rotation of the shaft can cause the change of the pitch angle. This is the result of the coupling component of the cone error, according to the previous analysis.

It can be seen that the head angle error is about 2.5′ from the Figure 7b; the roll angle error is about 1.86′ from the Figure 7c; and the pitch angle error is about 1.6′ from the Figure 7d.

#### 6.2.2. The Data Analysis after Compensation

**Figure 8:**
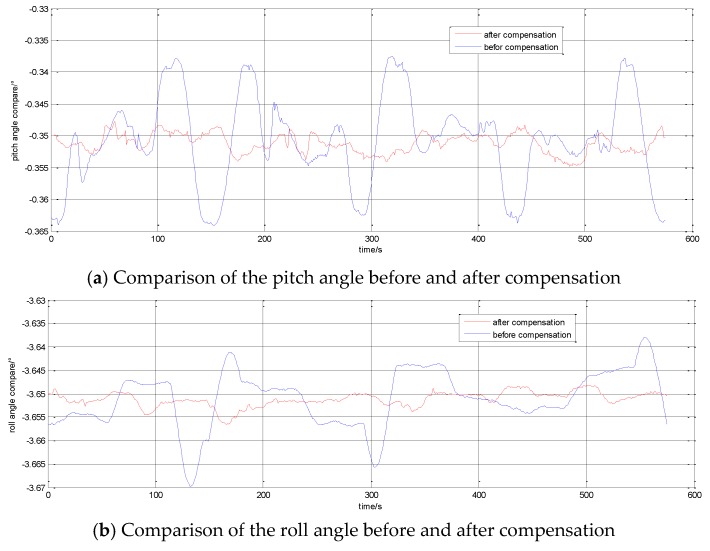

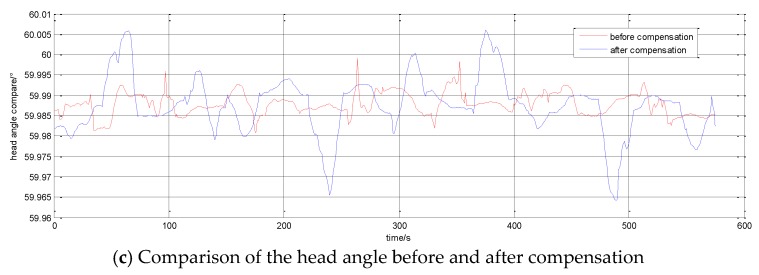
System attitude angle after compensating for the non-orthogonal angle and shaft swing angle.

After the shaft swing angle and the non-orthogonal angle are compensated for by using the methods mentioned in Section 5, the attitudes of INS are shown in Figure 8. The attitudes before the compensation are also shown in Figure 8.

Through data analysis, the changes of the attitude angle error before and after compension are as follows:

The head angle error reduces from 2.5′ to 49.32″; the roll angle error reduces from 1.86′ to 30.24″; and the pitch angle error reduces from 1.6′ to 25.2″.

There are still some changes of the attitude angles after calibrating the shaft swing angle and the non-orthogonal angle. This is because the shaft swing angle is not a constant. Therefore, the smaller the change of the swing angle, the higher the attitude accuracy of the system.

## 7. Conclusions

In this paper, we found that the shaft swing angle and the non-orthogonal angle are inevitable errors after analyzing the structure of the dual-axis rotation INS, and they are also coning errors. They not only can reduce the accuracy of the attitude, but can also reduce the position accuracy of the dual-axis rotation INS in long-term navigation. In order to improve the attitude accuracy further, a calibration method is proposed in this paper. Specifically, the following contributions have been made:

The modulation average frame $\bar{P}$ is established. The attitude transformation matrix $C_{b}^{n}$, attitude, and its error equations including the shaft swing angle and non-orthogonal angle are mainly derived in the frame $\bar{P}$. The static Kalman filter is used to estimate the coning angles (α_1_,α_2_), initial phase angles (ϕ_1,_ϕ_2_), and non-perpendicular angle (η).

Through the simulation and the static verification of the actual system, the results show that the calibration method is feasible. The attitude accuracy increased about 20%–30% after the precise compensation. The attitude error equation is reasonably simplified and the calibration method is accurate enough.

At the same time, we found that there are still slight perturbations of the attitude angle curve. This is caused by two factors; one is the encoder reading angle, and the other is the shaft swing angle that is not a fixed value. Furthermore, the calibration is established under the static condition and is not feasible for a ship at the docking side. For a wider range of applications, the static Kalman filter based on disturbed specific forces should be designed to identify the model coefficients.

## Figures and Tables

**Table 1:** Specifications of the designed dual-axis roational INS.

Characteristics	Description
Output rates	200 Hz
Accel. fixed bias (1σ)	100 μg
Accel. stochastic error	50 μg/h^1/2^
Accel. scale factor error	<20 ppm
Accel. misalignment error	<5′′
Range of accel	±40 g
Gyro fixed bias (1σ)	0.005°/h
Gyro stochastic error	0.0005°/h^1/2^
Gyro scale factor error	<20 ppm
Gyro misalignment error	<5′′
Gyro g-dependent bias	0.0001°/h/g
Range of Gyro	±300°/s
A/D resolution	32 bit
Supply Voltage	24 V, ±5 V
Turntable (1σ)	12′′

**Table 2:** Specifications of the designed dual-axis roational INS.

Parameter	Set Value	Calibrated Value	Calibrated Error
$\alpha_{1}$	5′	4′58.98′′	1.02′′
$\varphi_{1}$	1°	0°59′56.76′′	−3.24′′
$\alpha_{2}$	10′′	9′58.57′′	1.43′′
$\varphi_{2}$	1°	0°59′57.87′′	−2.13′′
η	20′′	18.39′′	1.61′′

**Table 3:** Calibration errors of simulations.

Parameter	Error Mean	Standard Deviation Error
$\alpha_{1}$	0.98′′	0.0238′′
$\varphi_{1}$	1.14′′	0.6103′′
$\alpha_{2}$	0.78′′	0.0078′′
$\varphi_{2}$	0.65′′	0.0317′′
η	1.05′′	0.0091′′
